# Genetic mapping reveals a marker for yellow skin in watermelon (*Citrullus lanatus* L.)

**DOI:** 10.1371/journal.pone.0200617

**Published:** 2018-09-28

**Authors:** Junling Dou, Xuqiang Lu, Aslam Ali, Shengjie Zhao, Lei Zhang, Nan He, Wenge Liu

**Affiliations:** 1 Zhengzhou Fruit Research Institute, Chinese Academy of Agricultural Sciences, Zhengzhou, Henan, China; 2 College of Horticulture and Forestry Sciences, Huazhong Agricultural University, Wuhan, Hubei, China; Huazhong Agriculture University, CHINA

## Abstract

As a diverse species, watermelon [*Citrullus lanatus* (Thunb.) Matsum. &Nakai var. lanatus] has different kinds of fruit sizes, shapes, flesh colors and skin colors. Skin color is among the major objectives for breeding. Yellow skin is an important trait in watermelon, but the underlying genetic mechanism is unknown. In this study, we identified a locus for yellow skin through BSA-seq and GWAS. A segregation analysis in F_2_ and BC_1_ populations derived from a cross of two inbred lines ‘94E1’(yellow skin) and ‘Qingfeng’(green skin) suggested that skin color is a qualitative trait. BSA-seq mapping confirmed the locus in the F_2_ population, which was detected on chromosome 4 by GWAS among 330 varieties. Several major markers, namely, 15 CAPS markers, 6 SSR markers and 2 SNP markers, were designed to delimit the region to 59.8 kb region on chromosome 4. Utilizing the two populations consisting of 10 yellow and 10 green skin watermelons, we found a tightly linked functional SNP marker for the yellow skin phenotype. The application of this marker as a selection tool in breeding programs will help to improve the breeder’s ability to make selections at early stages of growth, thus accelerating the breeding program.

## Introduction

Skin color is predominantly an extremely important characteristic for markets and breeders point of view, displaying wide range of variations. In the past, Many QTLs (quantitative trait locus) and genes related to fruit skin have been detected or cloned in different crops, such as apple, bitter gourd, grapefruit, pear, and melon [1–5]. Coloring pigments imparts different color or coloring schemes to the plant organs, one of them is anthocyanin. Anthocyanin synthesis in apple flowers is controlled by two *MYB* genes, two *UFGT* genes, one *bHLH3* gene and one *bHLH33*, while the *MdMYB1a*, *MdMYB1*, *MdbHLH3-1*, *MdbHLH33-1*, *MdUFGT2-1*, and *MdUFGT4* genes were disclosed to synthesize anthocyanin in apple skin. However, they may be involved in other plant functions at different fruit development stages in different cultivars [4]. In tomato, the study demonstrated that the *MYB12* transcription factor plays an important role in regulating the flavonoid pathway in tomato fruit and suggested strongly that *SlMYB12* is a likely candidate gene for the yellow color [6]. Strong relation existed between expression of these genes and skin color validated by transgenic approaches [7, 8]. Similarly, dominant and additive effect was under the control of more than three genes in bitter gourd. In another study, inheritance of skin color is hypothesized to be a duplicate dominant epistasis rather the dark green dominance over light green. Recessive form of *g-1* and *g-2* genes control light green skin. Genotype with dominant allele will result in the production of solid dark green skin. [5]. In another study, fruit shading enhances skin color, carotenes accumulation and chromoplast differentiation in grapefruit, Transcript levels of most of the genes (*PSY*, *PDS*, *ZDS1*, *βLCY1* and *βCHX*) enhanced during fruit ripening and were notably higher in light-grown fruits [3]. In Japanese pears, Inoue et al [9] screened 200 Random Amplified Polymorphic DNA (RAPD) markers in two cross breeds and eventually identified a RAPD marker to distinguish the green skin, which explained approximately 92% of the phenotypic variation in the green skin. *PyMYB10* plays an important role in the color formation of red pear skin, it is up-regulated in both the light-colored pear ‘Zaobaimi’ and the well-colored red pear ‘Yunhong-1’ [1]. In melon, research results demonstrate that the Kelch domain-containing F-box protein coding gene (*CmKFB*), which plays a key role in controlling skin and leaf color, functions as a post-transcriptional regulator, which diverts flavonoid metabolic flux. A metabolite analysis indicated that downstream flavonoids accumulate together with naringenin chalcone, while *CmKFB* expression diverts the biochemical flux towards coumarins and general phenylpropanoids [2].

Watermelon [*Citrullus lanatus*(Thunb.)Matsum.&Nakai], a member of cucurbitacea family, is globally famous and liked because of its high nutrition, flavor and aroma accompanied with divergent shapes and colors of flesh and skin. Haploid genome of watermelon has approximate size of 425 Mb (2n = 2X = 22) with 11 chromosomes. The genome sequence and the gene annotation of watermelon was released in 2013, following cucumber and melon [10–12] (http://cucurbitgenomics.org/), making it worthwhile to apply next-generation sequencing (NGS) to reveal the functions of genes by fine mapping. Similar to other cultivated cucurbits, watermelon has substantial divergence in various fruit traits. Watermelon has gained integral place in human diet because of its shelf life, skin color, nutritional value and they are its major quality parameters that are presumably concerned in research. Until now, some of these have been investigated at the molecular level, including seed color, seed size, fruit shape, flesh color, and skin color [13–15].

Divergent skin color and patterns in watermelon are preference of consumers that makes it commercially important and considerable importance has also been given to its esthetic value [8]. Watermelon breeders always focused on improving the cultivars quality by introducing novel traits in existing germplasm, one of the quality parameters is skin color that includes distinct skin patterns namely, solid, striped and intermittent [16, 17]. Previously, many studies hypothesized the inheritance pattern of skin color in watermelon. In the model demonstrated by Weetman [18] and Poole [19], it explains how striped and non-striped pattern are ascertained by three alleles at a single locus. Moreover, Weetman [18] confirmed that two loci account for the inheritance of striped patterns and skin color. This fact was further strengthened in another study by Kumar and Wehner [17] showing that two loci account for the solid dark green skin color. Primarily, green color skin prevailed in watermelon [16]. During the 1900s, in Japan and the United States, watermelon breeders produced new hybrids with a characteristic appearance of a distinct color and pattern. Unfortunately, they were not maintained, leading to the unavailability of these cultivars at present. However, the *p* allele in ‘Japan 6’ imparted thin and fainted lines on the skin, and ‘China 23’ had striped skin coupled with a medium green-colored network controlled by the *P* allele. ‘Long Iowa Belle’ and ‘Round Iowa Belle’ are characterized by irregularly distributed and randomly shaped whitish to green spots on a solid dark green skin and are determined by the *m* gene [18]. Golden yellow skin color in the ‘Royal Golden’ is attributed to inheritance of single recessive gene *go* [20]. Kumar and Wehner [17] suggested that the solid dark green skin color is controlled by two loci. Inheritance of solid dark green skin versus light (gray) skin showed duplicate dominant epistasis. Duplicate dominant epistasis gives rise to a 15:1 ratio (solid dark green: light skin pattern) in the F_2_ generation. When both the loci are homozygous recessive, we observe a light skin pattern. The *g*^*-1*^ and *g*^*-2*^ genes control the light green skin when in the homozygous recessive form [17]. Recent studies, enlightened the inheritance of foreground stripe pattern*D* (depth of skin color); and *Dgo* (background skin color) claiming to be governed by three independent loci [21]. Golden yellow fruit is inherited as a single recessive gene *go* [20] derived from ‘Royal Golden’ watermelon. The immature fruit have a dark green skin, which becomes more golden yellow as the fruit matures. The stem and older leaves also become golden yellow [16].

Recently, yellow skin watermelon has gained more popularity among consumers, and the market also appears to have a number of different yellow skin varieties, but information regarding the inheritance of the yellow skin trait is elusive and candidate genes controlling yellow skin in watermelon are not reported yet. In this study, we investigated the inheritance the pattern of the yellow skin trait in the F_2_ population of ‘94E1’ (yellow skin) ×‘Qingfeng’ (green skin). We identified a locus and linkage marker on chromosome 4 that was associated with watermelon yellow skin through genome wide association studies (GWAS) of 330 watermelon accessions and bulked-segregant analysis (BSA) by genotyping a pair of bulked DNA samples from two sets of individuals representing opposite extreme phenotypes (yellow individuals and green individuals). To our knowledge, this is the first report about the mapping and inheritance mechanism controlling watermelon yellow skin. The current research will help to shorten the breeding period for the yellow skin trait in watermelon. This study will not only accelerate the breeding process, but also provide valuable research tools to unravel the inheritance patterns of yellow skin in watermelon that will be helpful in the future.

## Materials and methods

### Plant materials

**Fig 1:**
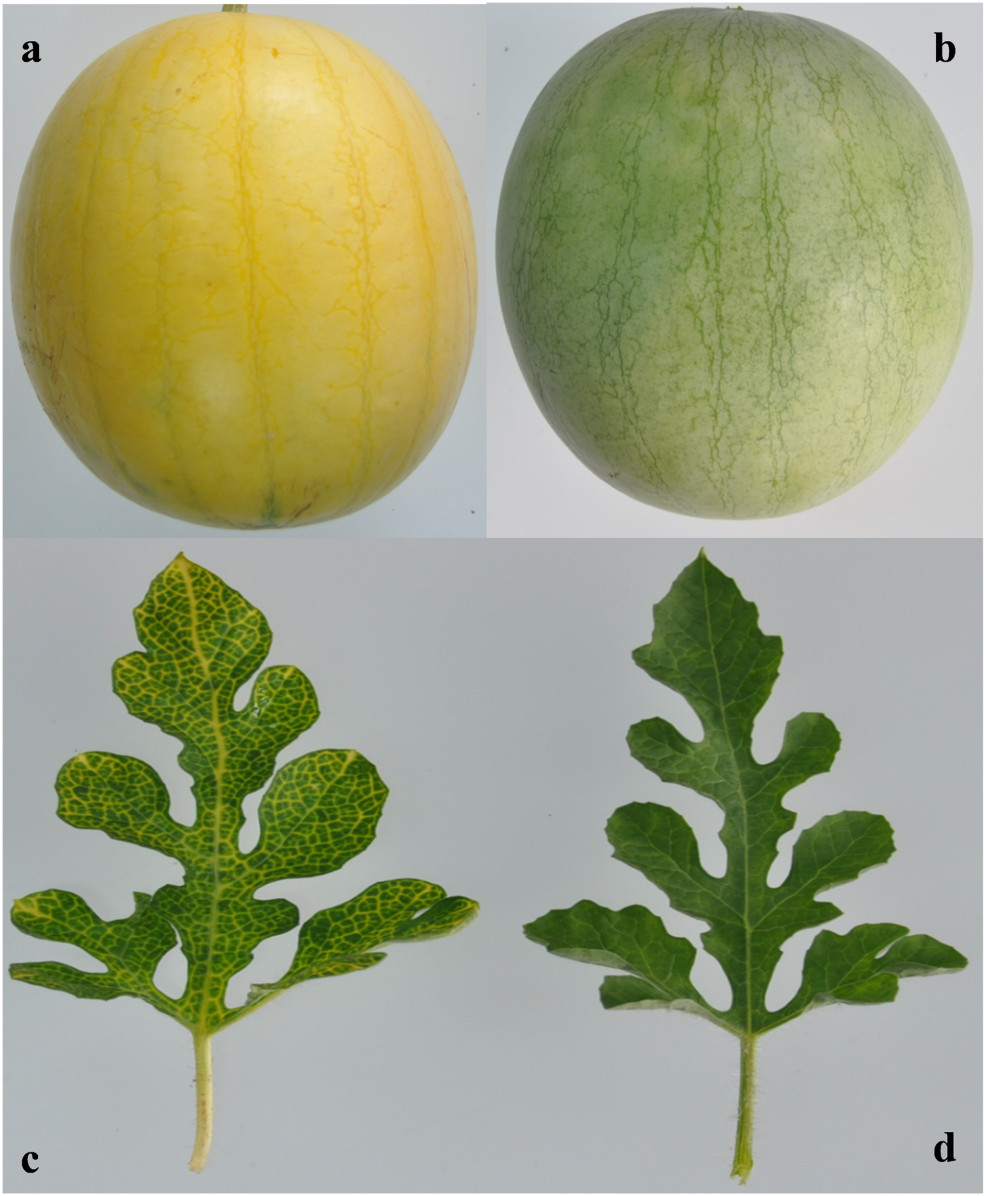
Fruit and leaf sample with two watermelons with yellow and green skin color. a, the fruit of yellow skin watermelon ‘94E1’; b, the fruit of green skin watermelon ‘Qingfeng’; c, the leaf of yellow skin watermelon ‘94E1’; d, the leaf of green skin watermelon ‘Qingfeng’.

The F_2_ population was raised by crossing two inbred lines, namely, ‘94E1’ (P_1_), a yellow skin cultivar having a characteristic appearance of yellow veins and petioles, and a green skin watermelon inbred line ‘Qingfeng’ (P_2_), with the green skin cultivar having a characteristic appearance of green leaves (Fig 1). A back cross population was obtained by hybridizing F_1_ with each parent to create BC_1_P_1_ (F_1_×94E1) and BC_1_P_2_ (F_1_×Qingfeng).

For segregation analysis, the F_2_ population was grown in two experiments in three seasons, Spring 2015, Autumn 2015 and Spring 2016, consisting of 897, 428 and 618 F_2_ individuals, respectively. The BC_1_P_1_ population (50 plants) and the BC_1_P_2_ (245 plants) population were investigated only in the Spring of 2016. In this study, there were only two types of fruit skin color: yellow and green, which was easily distinguished. For each plant, the phenotype was recorded (Table 1). All the materials were grown in the experimental area of the ‘Xinxiang’, Zhengzhou fruit research institute (Zhengzhou, China).

**Table 1:** Segregation of the F_2_ and BC_1_ populations.

Populations	Yellow skin	Green skin	Segregation ratio	Chi-square	P
Spring 2015 F_2_	676	221	3:1	0.063	0.80
Autumn 2015 F_2_	317	111	3:1	0.199	0.66
Spring 2016 F_2_	458	160	3:1	0.261	0.61
BC_1_P_1_	50	0			
BC_1_P_2_	126	119	1:1	0.200	0.65

### Bulked-segregant DNA analysis

Genomic DNA was isolated using the CATB method [22] from fresh leaves of the F_2_ plants in the Spring 2016 experiment, which were used for both the QTL-seq and CAPS markers analyses. Two DNA pools, the yellow skin pool (Y-pool) and green skin pool (G-pool), were constructed by mixing an equal amount of DNA from 30 yellow skin watermelon plants and 30 green skin watermelon plants. Pair-end sequencing libraries (read length 100 bp), with insert sizes of approximately 500 bp, were prepared for sequencing with an Illumina Genome Analyzer IIx machine. The short reads from the Y-pool and G-pool were aligned to the ‘97103’ reference genome [12] with the BWA software [23]. First, alignment files were converted to SAM/BAM files using SAM tools [24] and were then applied to the SNP-calling filter ‘Coval’, which was previously developed [25] to increase SNP-calling accuracy. The SNP-index was calculated for all the SNP positions. We excluded SNP positions with a SNP-index of <0.6 and a read depth <6 from the two sequences, as these may represent spurious SNPs called due to genomic repeat sequence, sequencing or alignment errors.

Two parameters, the SNP-index and Δ (SNP-index) [25, 26], were calculated to identify candidate regions for the watermelon yellow skin QTL. An SNP-index is the proportion of reads harboring the SNP that is different from the reference sequence. Δ (SNP-index) was obtained by subtracting the SNP-index of the Y-pool from that of the G-pool. Thus, the SNP-index = 0 if the entire short reads contained genomic fragments from a mutation line; and the SNP-index = 1 if all the short reads were from the reference genome ‘97103’. An average of the SNP-indexes of the SNPs located in a given genomic interval was calculated using a sliding window analysis with a 1 Mb window size and a 10 kb increment. The SNP-index graphs for the Y-pool and G-pool, as well as the corresponding Δ(SNP-index) graph, were plotted.

To generate confidence intervals of the SNP-index value under the null hypothesis of no QTL, we carried out a computer simulation. We first made two pools of progeny with a given number of individuals by random sampling. From each pool, a given number of alleles corresponding to the read depth were sampled. We calculated SNP-index for each pool and derived Δ(SNP-index). This process was repeated 10, 000 times for each read depth, and confidence intervals were generated. These intervals were plotted for all the genomic regions that had variable read depths.

### GWAS

A total of 330 watermelon accessions, which contained different fruit skin colors were re-sequenced, and 2.3T of data, which had an 85.42% average genome coverage and a 9.24×average sequence depth, were obtained. The watermelon accessions were genotyped using 4,661,625 evenly spaced SNPs. The association between fruit skin color and each SNP was tested using a unified mixed model [27, 28]. This mixed linear model included principal components [29] as fixed effects to account for the population structure and a kinship matrix [30] to account for familial relatedness. Utilizing the Bayesian information criterion, a backward elimination procedure was implemented to determine the optimal number of principal components to include in the mixed model [31]. The false discovery rate was controlled at 5% using the Benjamini and Hochberg [32] procedure. A likelihood ratio–based r2 statistic was used to assess the goodness of fit of each SNP [33]. All the analyses were conducted using the Genome Association and Prediction Integrated Tool software package [34].

### Fine mapping through the CAPS markers

The watermelon genome sequence was obtained from the watermelon database (http://www.icugi.org), and the sequence was compared with the re-sequencing data to identify SNPs via a filter pipeline [26]. To minimize the genetic interval for fine mapping and to verify the accuracy of the BSA-Seq, 15 cleaved amplified polymorphic sequences (CAPS) markers for the SNPs generated from the BSA-seq, 6 SSR markers and 2 SNP markers were developed for genotyping (Table 2).

**Table 2:** Information on the major markers in chromosome 4 used to analyze the polymorphisms.

Marker Number	Primer Sequence	position	Digest(s)
CAPS01	F—AGTTCAATGACCAAAGGTGAACT R—AATGTCACGTTGTCATGTCCA	Chr4:59846	BglII(a/gatct):445+206,651
CAPS02	F -TGCAACTACAGGTGTAAGGCA R—CCAACAACCATTCACGCAGG	Chr4:67981	SalI(g/tcgac):445+317,762
CAPS03	F—ACCTTGGGAGCCCTTAGAGA R—AGGTAGAACACTCAAACTTAGGGT	Chr4:137311	HapII(c/cgg):335+316,651
CAPS04	F—GAACGTCCCAAGGAACCACA R—TCCAACTTCAAACCCTTCCTCT	Chr4:840557	TaqI(t/cga):372+352,724
CAPS05	F—CGTACGTATACCACCACCCG R—TGGTCATCGTCATCGCTAAGG	Chr4:909302	EcoRI(g/aattc):405+353,758
CAPS06	F—TGACCTATTCTCGCTTGCCC R—TCCCCTTGCATTTTCCCTCG	Chr4:999594	Eco32I(gat/atc):437+292,729
CAPS07	F—AATAGCTCTTCGTTCATGTACTCT R—AGACATCACGTGGAAATTAGAAG	Chrr4:3362878	HapII(c/cgg):330+328,658
CAPS08	F—ACCCTACCATGTCTATTAACCATCC R—AGGAAACAATTGTATAGCTATGGTTT	Chr4:3848297	HindIII(a/agctt):471+252,723
CAPS09	F—AGTGTTCAAATTAACCAAGGGGA R—ACTCGCATGTCACCTACACG	Chr4:3874492	HindIII(a/agctt):458+252,710
CAPS10	F—TGACTACCAATCAGACAAATCTTGA R—GGTGGTCAGTGGGAATTGCA	Chr4:4156897	TaqI(t/cga):366+312,678
CAPS11	F—ACATCATCTCTCCCGCTCCA R—TGTTGCGTTTAGGGTTGGTT	Chr4:4994766	AluI(ag/ct):473+179,652
CAPS12	F—ACCGCTAAAAACTAAATGAGTGTT R—AGCATGCAAAATAACGCCCG	Chr4:5533196	AsuII(tt/cgaa):714,357+357
CAPS13	F—ATCAATTATGCCTCAATAGGTTTCTT R—TGGAGTATTGCATTGAGTAGCA	Chr4:6486343	BfaI(c/tag):494+186,680
CAPS14	F—TTCCTTGCAGCCAACTCACT R—CAAGGGTGCTCGAGAAGGTT	Chr4:6838095	EcoRI(g/aattc):782,492+290
CAPS15	F—TCCTTCAACAGTTGGCGACT R—CGCCTTGAGTGGGCTATAGG	Chr4:6862096	AluI(ag/ct):408+281,689
SNP02	F—TGCATGATGAGCCTTCTTTGAA R—TAGACGGGGCTCACAAGTCA	Chr4:48456	
SNP03	F—AGAATCAACATGAGAATGCAACA R—ATCAAAGGACGAGCCCTCAC	Chr4:37932	
SSR01	F—GCATATCTAATCTATGAGCACCTAC R—ACAATATTTTGTTGTAGAGTAGAGGA	Chr4:74647–75150	
SSR02	F—GGGTTTGTTTCCATTTCCCT R—GGAAGGGTTCTGCATGTGTT	Chr4:328582–328860	
SSR03	F—GGGCCTGTGATCCATAGAGTAAAA R- GATGCATGCTTAAATTCTTATTTCTTCA	Chr4:1572774–1572919	
SSR04	F—GGATGGAAAAATTGGAAAGAA R—TGAGGCGACTGTGGTTCATA	Chr:3199559–3199824	
SSR05	F—ATGCTTAGTAGGCGGGTTTC R—TGCCGGAGATTTTAGACTGT	Chr4:3526134–3526604	
SSR06	F—AATGGTTGCGTTGAAGTTCC R—TTGGCATCCTCTTCTTCTTCA	Chr4:5959998–5960201	

The PCR was carried out in a total volume of 10 μL containing 5 μL of 2× Power Taq PCR Master Mix (BioTeke, China), 0.5 μL of 10μM per primer, 1μL of 200 ng of genomic DNA and 3μLof RNase-free water. All the amplifications were performed on an EasyCycler (Analytik Jena, Germany) under the following conditions: 5 min at 95°C; 28 cycles of 30 s at 94°C; 30 s at 56°C; 50 s at 72°C; and a final extension step at 72°C for 10 min. The amplified PCR products were digested using suitable restriction endonucleases according to the manufacturer’s instructions at 37°C or 65°C for 4–10 h. The digests were resolved by electrophoresis on a 1.0% agarose gel or PAGE gel and were visualized on a Versa Doc (Bio-Rad) after staining with ethidium bromide (EB).

### Real-time PCR analysis

The ovaries and fruit skin from different developmental stages were collected for RNA extraction to check the gene expression. Total RNA was extracted using the plant total RNA purification kit (GeneMark, China) following the manufacturer's instructions. The cDNA was synthesized with reverse transcriptase M-MLV (RNase H-) following the manufacturer’s instructions (Takara, Japan).

The primers for the genes and the reference gene *Actin* [35] used in quantitative reverse transcription polymerase chain reaction (qRT-PCR) were designed based on Cucurbit Genomics Database (http://www.icugi.org). The expression levels of the target genes were evaluated by qRT-PCR using a LightCycler480 RT-PCR system (Roche, Swiss). All the reactions were performed using the SYBR Green real-time PCR mix according to the manufacturer’s instructions. Each 20 μL RT-PCR reaction mixture containing 1 μL of cDNA, 1 μL of forward primer (10μM), 1 μL of reverse primer (10μM), 10 μL of 2×SYBR Green real-time PCR mix, and nuclease-free water to final volume of 20 μL, was preheated at 95 °C for 5 min, followed by 45 cycles of 95 °C, 60 °C and 72 °C for 30 sec. High Resolution Melting was performed under the following conditions:1 min at 95 °C; 1 min at 40 °C; 1 s at 65 °C; and continuous at 95 °C. All the experiments were performed in triplicate. The raw data from the qRT-PCR were analyzed using LCS480 software 1.5.0.39 (Roche, Swiss) and the relative expression was determined using the 2^-ΔΔCT^ method [36].

### Testing markers for linkage to the yellow skin of watermelon

All the CAPS markers, SSR markers and SNP markers that met the criteria for putative linkage to yellow skin were selected for testing across 618 F_2_ individuals in Spring 2016. If linkage was observed, the markers were tested in 20 germplasm resources, which contained 10 different yellow skin watermelons and 10 different green skin watermelons.

## Results

### Inheritance of yellow skin in watermelon

The segregation ratio of yellow skin among the three F_2_ populations in 2 years is presented in Table 1. The variation in the five segregating populations (F_2_—2015 Spring, F_2_—2015 Autumn, F_2_—2016 Spring, BC_1_P_1_-2016 Spring, BC_1_P_2_-2016 Spring) showed that the yellow skin alleles were dominant over the green skin alleles. The segregation ratio computed by the chi-square test for these three populations was in accordance with 3:1 (Table 1), suggesting that watermelon yellow skin was a qualitatively inherited trait controlled by a single dominant gene. Interestingly, the leaves, veins and petioles all showed a green color at the seedling stage. However with the development of the fruit, the veins and petioles of the yellow skin watermelon individuals gradually turned yellow, which made it very easy to distinguish the yellow watermelon from the green watermelon (Fig 1).

### BSA-seq reveals that the watermelon yellow skin gene is located on chromosome 4

**Fig 2:**
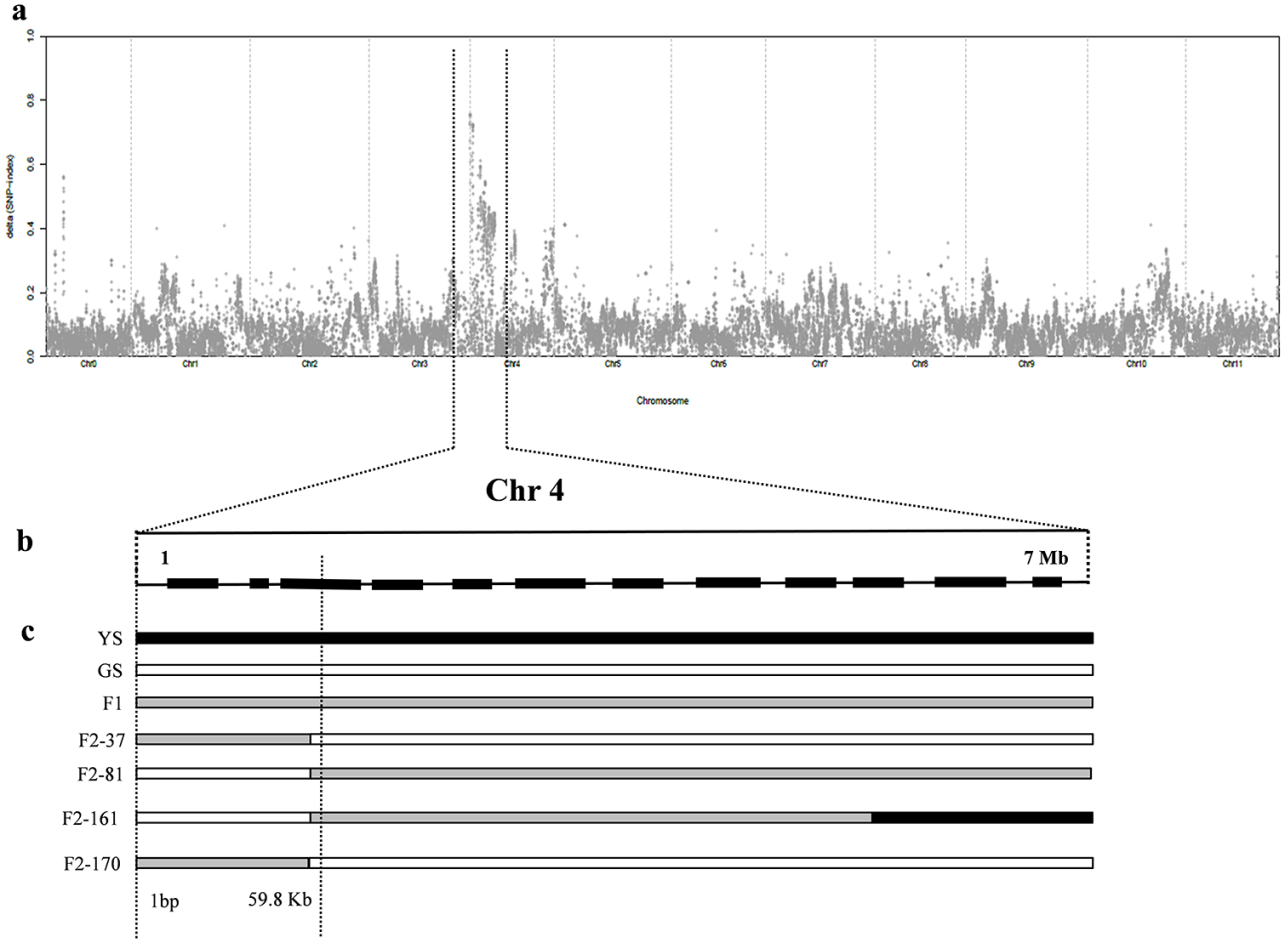
Fine mapping of the watermelon yellow skin trait. a: Δ (SNP-index) graph from the QTL-seq analysis; b: The yellow skin was identified to an interval of 1–7 Mb on chromosome 4. c: The candidate gene was narrowed to a 59.8 kb interval according to 4 recombine individuals in F_2_ population. YS: yellow skin; GS: green skin. Yellow skin was dominant.

A total of 30 yellow skin individuals (Y-pool) and 30 green skin individuals (G-pool) were selected from the two populations planted in Autumn 2015. We sequenced the two pools using the Illumina HiSeqTM PE150 platform. A total of 33.753 GB of raw data was generated for both pools, with approximately 30× depth and more than 99% coverage for each. The data was aligned to the ‘97103’ watermelon reference genome (http://www.icugi.org), and 247,012 SNPs were identified between the two pools. Each identified SNP was used to compute the SNP-index. The graph for the Δ (SNP-index) was plotted and computed against the genome positions by combining the information of the Y-pool and G-pool SNP-index (Fig 2a). The regions on chromosome 4 from 1 bp to 7 Mb had an average higher than 0.6 Δ (SNP-index) (Fig 2a and 2b) and significantly differed from 0 of the Δ (SNP-index) value at a 90% significance level. The result showed that there was a candidate gene controlling the yellow skin trait at this region (S1 Table).

### GWAS identify watermelon yellow skin gene located on chromosome 4

A GWAS was performed for watermelon fruit skin color, which utilized a total 330 watermelon accessions, including different skin colors to capture the maximum genetic diversity, and was genotyped with a high-density, genome-wide coverage of 4,661,625 evenly spaced SNPs. To reduce the incidence of false-positive signals, a unified mixed linear model that controls the population structure, and familial relatedness was used (6PC+K [for 6 Principal Components and Kinship]; [27]) to test the associations between fruit skin color, and 534,162 of the 4,661,625 SNPs showed minor allele frequency > 5%.

**Fig 3:**
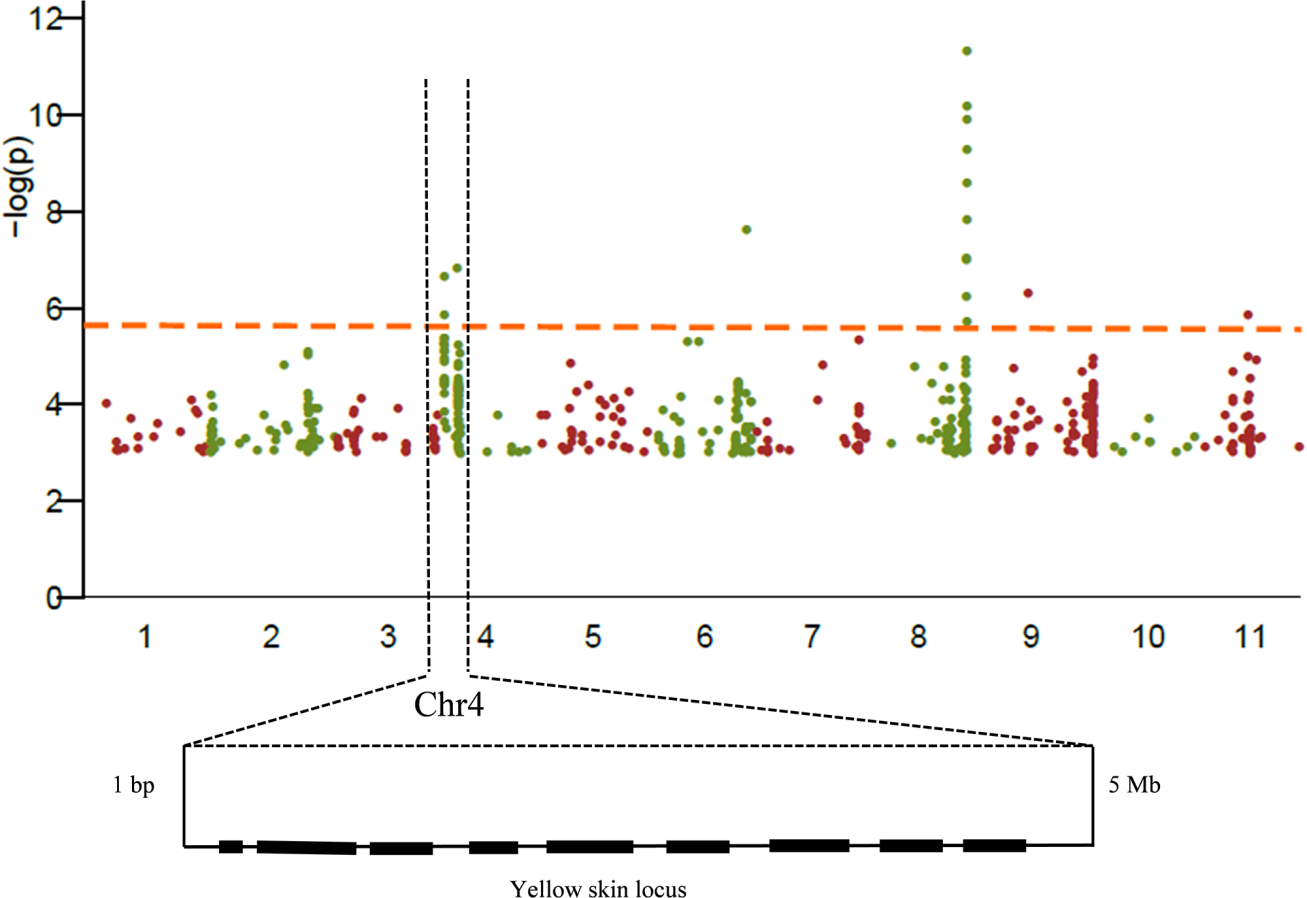
The locus for watermelon yellow skin was identified through GWAS. Manhattan plot of the genome-wide association for watermelon skin color showed that the region of 1–5 Mb on watermelon chromosome 4 controlled yellow skin.

A difference of 10-fold was calculated through the analysis of the natural variation of watermelon yellow skin across the diversity panel coupled with an 85% (broad-sense) heritability, revealing that the observed natural variation was largely dictated by the genetic variation across the population rather than environmental factors [37, 38]. The GWAS profiles among the 330 varieties identified a major locus of 1 bp—5 Mb designated on watermelon chromosome 4, which may control watermelon yellow skin (Fig 3, S2 and S3 Tables).

### Analyses of the markers narrowed down the watermelon yellow skin gene to a 59.8 Kb interval

To confirm the yellow skin locus detected by the BSA-seq and GWAS, we conducted a polymorphic analysis of 428 F_2_ individuals from the Autumn 2015 experiment. We developed 15 CAPS markers, 6 SSR markers and 2 SNP markers from chromosome 4 (Table 2). Individuals from F_2_ were subjected to the polymorphism analysis, and an interval was identified to control watermelon yellow skin, which was physically located in the region of 1 bp– 59.8 kb on chromosome 4 (Fig 2c). In this region of reference genome ‘97103’, there are many gaps, only one gene (*Cla002755*) was found, and it has no functional annotation. We blasted this region to the reference genome ‘Charleston Gray’ (http://www.icugi.org) and also found many gaps. The same sequence of the *Cla002755* gene existed in this region.

### Sequence and expression analysis of *Cla002755*

The DNA sequence of *Cla002755* has a total length of 237 bp, without introns, and starts from the 7th base on the forefront of chromosome 4. The DNA sequences of *Cla002755* were obtained from the yellow skin watermelon ‘94E1’ and green skin watermelon ‘Qingfeng’ parents. While no sequence changes were found between the two parents. The expression pattern of *Cla002755* was investigated using RT-PCR in the fruit skin at different developmental stages in the yellow and green skin parents. During fruit development, the expression of *Cla002755* was not significantly different between the two parents. Thus, this further suggested that *Cla002755* may not be the candidate gene controlling the yellow skin trait.

### A SNP marker is linked to watermelon yellow skin

**Fig 4:**
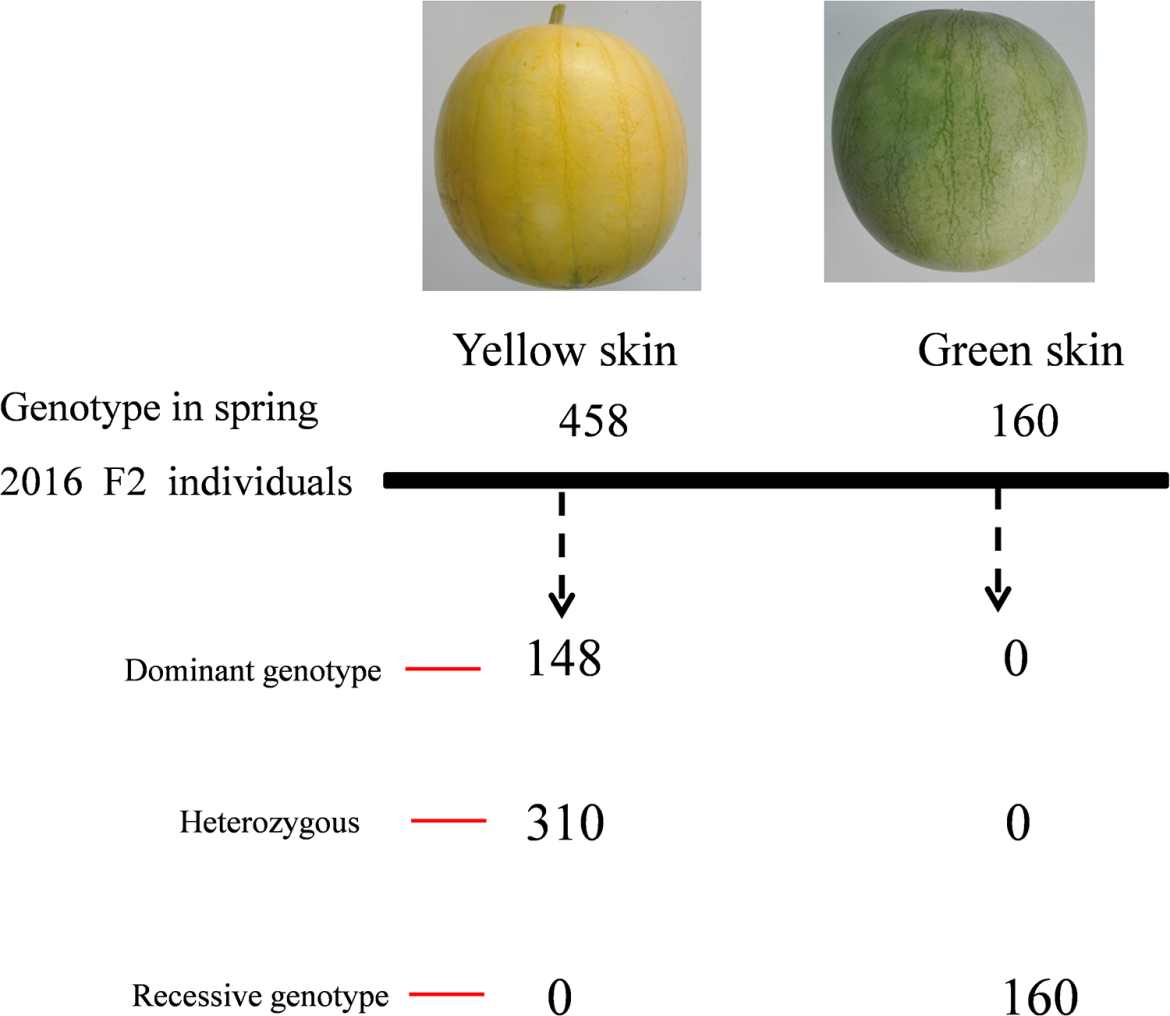
Co-segregation of the yellow skin phenotype and the marker SNP02 in the F_2_ population. Genotyping by PCR revealed that 148 yellow skin individuals were dominant homozygous and the 310 yellow skin individuals were heterozygous, whereas 160 green skin individuals were recessive homozygous.

We developed 15 CAPS markers, 6 SSR markers and 2 SNP markers to analyze the linkage to yellow skin. Fortunately, we found the SNP02 marker for marker-assisted selection of yellow skin in watermelon, which was identified in 618 F_2_ population individuals from Spring 2016 (Fig 4). Using this maker, we screened a population, consisting of 10 yellow skin and 10 green skin watermelons. As expected, the genotypes perfectly matched the phenotype of the yellow and green skin watermelon. These results indicated that the SNP02 marker was tightly associated with the watermelon yellow skin trait.

## Discussion

With the advancement in sequencing technology, the NGS along with recently drafted reference genome will expand our skills and will provide us new directions to unveil facts of linkage map. However, population size is limited and it is high priced technology for gene mapping, because cost of production increased when every individual plant has to be genotyped. NGS-assisted BSA is less laborious, much cheaper and has no population size limitation for genotyping work because it provides an effective and simple method to identify molecular markers linked to target genes/QTLs controlling traits by genotyping only a pair of bulked DNA samples from two sets of individuals with distinct or opposite extreme phenotypes [26, 39]. Sometimes, determination of some complex quantitative trait accurately during genotyping is problem some. Yellow skin is a qualitative trait and is easy to distinguish from normal traits in watermelon. We determined the locus for yellow skin on chromosomes 4 by taking advantage of NGS-assisted BSA approach together with GWAS in watermelon.

Watermelon is an important crop and varies greatly in skin color, including black, dark green, light green, yellow and others [12]. Skin color has always been concerned trait from the consumer point of view in many fruits and vegetables; yellow skin watermelon has become preference for consumers because of its delightful appearance and high carotenoids contents. However, studies at genetic level are few or failed to explain the inheritance mechanism of yellow skin in watermelon. Previously, some researchers claimed that yellow skin in watermelon is controlled by a single recessive gene [20]. While in another study, they were contradictory results explaining that it is controlled by a single dominant gene [21]. In our two year study, we defined that a single dominant gene controls the yellow skin, resulting in yellow (AA and Aa) and green (aa) fruit. In the F_2_ population, all the yellow skin watermelons had yellow veins and petioles, suggesting that the yellow skin, veins and petioles may be controlled by the same gene, which is responsible for same type of pigment in the skin, veins and petioles.

No reports have been published until now about candidate gene controlling yellow skin in watermelon but some literature are available focusing the inheritance pattern of yellow skin. In this study, watermelon skin color was divided into yellow and green categories among the F_2_ population. The gene for yellow skin was localized to a single locus on chromosome 4. To identify the candidate gene, we performed a combinatorial approach integrating BSA-seq in a F_2_ population and GWAS in 330 watermelon accessions. The same locus on chromosomes 4 in the watermelon genome was mapped using the GWAS and NGS-assisted BSA approach (Figs 2 and 3). The BSA-seq was utilized for the genome-wide identification of SNPs between two bulked pools, which are used to develop molecular markers in gene mapping [40, 41]. The candidate gene was delimited in the region of 1 bp—59.8 kb on chromosome 4 using 15 CAPS markers, 6 SSR markers and 2 SNP markers (Fig 2c; Table 2). The sequence annotation analysis showed that there was a putative gene (*Cla002755*) in this region based on the watermelon reference genome ‘97103’ (http://www.icugi.org). The DNA sequence of *Cla002755* has a total length of 237 bp, without introns and starts from the 7th base on the forefront of chromosome 4. A large number of gaps in the 59.8 kb interval were found in the reference genome ‘97103’, and we blasted the interval to the reference genome ‘Charleston Gray’ and found many gaps, which presents only one gene homologous with *Cla002755*. Sequence alignments between the yellow skin plant and green skin plant revealed no sequence changes in *Cla002755* between the two parents. The predicted gene was not differentially expressed during fruit development and ripening. The results identified that *Cla002755* may not be the candidate gene for yellow skin watermelon.

Functional markers, where the genotypic sequence used for the selection is the cause of the phenotype, are the preferred marker types because there is no recombination between the marker and the trait gene [42]. Based on the 59.8 kb interval, an SNP marker (SNP02) was developed to assess the polymorphisms among 618 F_2_ population individuals in Spring 2016 and 20 watermelon accessions, including 10 yellow skin plants, which possess yellow veins and yellow petioles and 10 green skin plants. The results showed that the marker SNP02 was closely linked to yellow skin watermelon and could be used to distinguish yellow and green skin.

Selection, migration, and genetic drift are responsible for genetic variants and gene mutations that started as rare ones with a very low frequency and that occur in one or few individuals in a given population, and these rare variants turned into common ones with the passage of time. There is remarkable development in various crops and model plants in developing comprehensive maps of genome changes [43–46]. It has been reported that the watermelon genome speciation event occurred 15–23 million years ago [12]. Predominantly. germplasm of wild watermelon is comprised of green skin. Different skin colors resulted because of the progress of evolution, artificial selection and gene mutations. The mutation of the yellow skin gene may occur at the forefront region of chromosome 4 in watermelon genome. In this interval, there may be two or three closely linked genes that control the phenotype of yellow skin, yellow veins and yellow petioles. The mutation in this region not only causes the skin color variation of the fruit, but also is the reason for the variation of the phenotype in the veins and petioles. Leaf color mutations could affect plant photosynthesis and growth and development. In general, we consider that the yellowing of leaves may reduce plant photosynthesis and growth [47, 48]. However, in this experiment, the yellow veins and petioles did not show any affect. Therefore, we infer that this candidate region plays a very important role in controlling plant photosynthesis. This region was poorly assembled with a large number of gaps in the ‘97103’ and ‘Charleston Gray’ reference genomes at the present time (http://www.icugi.org). In the current study, we were unable to locate the candidate gene for yellow skin at the forefront region (59.8 kb) of chromosome 4. If there a better watermelon genome was assembled, perhaps we would have easily detected the candidate gene(s) for yellow skin, yellow vein and petioles in this region.

This study provides a good entry point to explain the genetic mechanisms of fruit development as well as provides fundamental insights into the domestication and selection history of watermelon. Though this study does not demonstrate the candidate gene of yellow skin, a tightly linked SNP marker was developed which can be used to identify the yellow skin watermelon at the seedling stage. Additionally, the current results will be useful in marker assisted breeding, and they will also be helpful in laying the foundation for watermelon breeding with high photosynthesis efficiency.

## Supporting information

S1 Table The raw data of SNP and SNP-index information of BSA on chromosome 4. (XLSX)

S2 Table The information of clean data and candidate genes about GWAS on chromosome 4. (XLSX)

S3 Table The position of high P-value on GWAS. (XLSX)
